# SIRT5‐mediated deacetylation of LDHB promotes autophagy and tumorigenesis in colorectal cancer

**DOI:** 10.1002/1878-0261.12408

**Published:** 2018-12-03

**Authors:** Liang Shi, Hui Yan, Shuxian An, Mengqin Shen, Wenzhi Jia, Ruixue Zhang, Li Zhao, Gang Huang, Jianjun Liu

**Affiliations:** ^1^ Department of Nuclear Medicine Renji Hospital School of Medicine Shanghai Jiao Tong University China; ^2^ Department of Cancer Metabolism Shanghai University of Medicine and Health Sciences China

**Keywords:** autophagy, colorectal cancer, deacetylation, lactate dehydrogenase B, sirtuin 5

## Abstract

Lactate dehydrogenase B (LDHB) is a glycolytic enzyme that catalyses the conversion of lactate and NAD ^+^ to pyruvate, NADH and H^+^. Protons (H^+^) generated by LDHB promote lysosomal acidification and autophagy in cancer, but how this role is regulated has not been defined. In this study, we identified an important post‐translational mechanism by which LDHB is regulated during autophagy in cancer cells. Mass spectrometry revealed that protein sirtuin 5 (SIRT5) is a binding partner of LDHB that deacetylated LDHB at lysine‐329, thereby promoting its enzymatic activity. Deacetylated LDHB increased autophagy and accelerated the growth of colorectal cancer (CRC) cells. Notably, SIRT5 knockout or inhibition by GW5074 increased LDHB acetylation at K329 and inhibited LDHB activity, which downregulated autophagy and CRC cell growth in vitro and in vivo. Clinically, the LDHB‐Ac‐K329 staining score in CRC tissues was lower than that in corresponding peritumour tissues. Low LDHB‐Ac‐K329 status was associated with malignant progression of human CRC and served as a potential prognostic indicator for patients with CRC. Altogether, we conclude that SIRT5‐induced deacetylation of LDHB triggers hyperactivation of autophagy, a key event in tumorigenesis. Thus, the SIRT5/LDHB pathway may represent a novel target for treating CRC.

**Abbreviations**
CRC: colorectal cancer
FAO: fatty acid oxidation
KO: knockout
LDHA: lactate dehydrogenase A
LDHB: lactate dehydrogenase B
MCT1: monocarboxylate transporter 1
OCR: oxygen consumption rate
PTM: post‐translational modifications
SIRT5: sirtuin 5
TCA: tricarboxylic acid
WT: wild‐type

## 1 Introduction

Colorectal cancer (CRC) is the third most common cancer and the second leading cause of cancer‐related death worldwide (Bray *et al*., 2018). Recent studies have shown that autophagy is robustly activated in many types of malignant tumours and contributes to the development and progression of cancers, including CRC (White, 2012). During the autophagic process, intracellular proteins, lipids and organelles are isolated to form an autophagosome (White, 2012). Then, the lysosome fuses with the autophagosome, and this process provides protons and acid‐activated proteases and allows cellular organelles to be broken down and reused for biosynthesis and energy metabolism (Maclean *et al*., 2008). Autophagy is activated in the intestinal epithelium of patients and in a mouse model of CRC (Levy *et al*., 2015). Autophagosomes are actively produced and promptly consumed in human CRC cell lines (Sato *et al*., 2007). Autophagy is essential for maintaining cellular homoeostasis and supporting cell survival and growth (Ryter *et al*., 2013). However, the underlying molecular mechanisms of autophagy in CRC remain elusive.

Lactate dehydrogenase B (LDHB) is a crucial glycolytic enzyme that catalyses conversion of lactate and NAD^+^ to pyruvate, NADH and H^+^ (Quistorff and Grunnet, 2011). A recent study has shown that LDHB plays an important role in autophagy in cancer cells (Brisson *et al*., 2016). LDHB interacts with V‐ATPase at the lysosomal surface; thus, H^+^ protons generated by LDHB directly promote lysosomal acidification and autophagy (Brisson *et al*., 2016). LDHB‐mediated autophagy benefits both oxidative and glycolytic cancer cells. In oxidative cancer cells, monocarboxylate transporter 1 (MCT1), which is highly expressed on the surface of oxidative cancer cells, facilitates the entry of lactate into tumour (Hui *et al*., 2017; Sonveaux *et al*., 2008). LDHB catalyses the oxidation of lactate to pyruvate, with the transformation of NAD^+^ to NADH, and H^+^. Pyruvate then fuels the tricarboxylic acid (TCA) cycle. H^+^ generated by LDHB promotes lysosomal acidification and autophagy which offers cytoprotection by recycling oxidized proteins and organelles when oxidative cancer cells face redox stress (Navarro‐Yepes *et al*., 2014; White, 2012). In glycolytic cancer cells, a large amount of intracellular lactate was catalysed from pyruvate by lactate dehydrogenase A (LDHA) (Shi *et al*., 2014). This provides sufficient substrate for LDHB. Autophagy sustains glycolytic cancer cell survival in metabolically restricted microenvironments by providing an additional source of energetic and biosynthetic precursors (Rabinowitz and White, 2010). LDHB‐mediated autophagy transfers protons to lysosomes and thus contributes to pH homoeostasis in glycolytic cancer cell (Spugnini *et al*., 2015). Thus, understanding the mechanisms that control LDHB‐mediated autophagy may help to identify new targets for treating CRC.

Sirtuin 5 (SIRT5) is a member of the NAD‐dependent sirtuin family that regulates acetylome signalling networks via post‐translational modifications (PTM) involving lysine deacetylation, desuccinylation, demalonylation and deglutarylation (Baur *et al*., 2010; Guarente and Picard, 2005; Yang *et al*., 2015). SIRT5 has been demonstrated to regulate metabolic enzymes by PTM and control several cellular metabolism pathways, including the TCA cycle and glycolysis (Nishida *et al*., 2015; Park *et al*., 2013), urea cycle (Nakagawa *et al*., 2009; Nakamura *et al*., 2012; Tan *et al*., 2014) and fatty acid oxidation (FAO; Colak *et al*., 2015; Rardin *et al*., 2013; Sadhukhan *et al*., 2016). Recently, SIRT5 was reported to be overexpressed in CRC tissues and to predict poor overall survival (Wang *et al*., 2018). Although one study has shown that SIRT5 regulates ammonia‐induced autophagy in human breast cancer cell lines MDA‐MB‐231 (Polletta *et al*., 2015), the functions of SIRT5 in autophagy in CRC remain largely obscure.

Herein, we report that SIRT5 is an important regulator of autophagy due to its interaction with LDHB. The underlying mechanism involves deacetylation modification and subsequent promotion of LDHB enzymatic activity. Our in vivo and in vitro data indicate that the specific deacetylation of LDHB by SIRT5 is essential for cancer development. Moreover, the lysine‐329 acetylation status of LDHB negatively correlates with the malignant progression of human CRC. These data provide a rationale for the use of a SIRT5/LDHB pathway as the potential target for therapeutic intervention in CRC with an abnormally activated autophagy status.

## 2 Materials and methods

### 2.1 Cells and reagents

HEK293T, HCT116 and DLD1 cells were purchased from ATCC and cultured in Dulbecco's modified Eagle's medium (DMEM) (Gibco, Carlsbad, CA, USA) supplemented with 10% fetal bovine serum (FBS; Gibco). Inhibitors: GW5074 (Selleck Chemicals, Houston, TX, USA), leupeptin (Sigma‐Aldrich, Burlington, MA, USA) and oxamate (Sigma‐Aldrich). Antibodies: SIRT5 (catalogue #15122‐1‐AP, Proteintech, Rosemont, IL, USA), Flag M2 (catalogue #F1804, Sigma‐Aldrich), HA (catalogue #H9658, Sigma‐Aldrich), Ki67 (catalogue #ab16667, Abcam, Cambridge, MA, USA), and cleaved caspase‐3 (catalogue #9661, Cell Signaling Technology, Boston, MA, USA), optineurin (catalogue #ab23666, Abcam), β‐tubulin (catalogue #66240‐1‐Ig, Proteintech), anti‐pan‐acetyl‐lysine (catalogue #9681, Cell Signaling Technology), anti‐pan‐succinylation (catalogue #PTM‐401, PTM Biolabs, Hangzhou, China), anti‐pan‐glutarylation (catalogue #PTM‐1151, PTM Biolabs), anti‐pan‐malonylation (catalogue #PTM‐901, PTM Biolabs), LDHB (catalogue #ab85319, Abcam) and LC3 (catalogue #CY5992, Abways, Shanghai, China). Antibody specifically recognizing acetylation at lysine‐329 of LDHB was prepared commercially at Shanghai HuiOu Biotechnology Co. Ltd (Shanghai, China). Synthesized peptide TLWDIQK(Ac)DLKDL was coupled to KLH as antigen to immunize rabbit. Anti‐serum was collected after five doses of immunization.

### 2.2 CRISPR–Cas9 knockout and re‐expressed cell lines

To generate HCT116/DLD1 SIRT5‐knockout (KO) and HCT116/DLD1 LDHB‐KO cell lines, the sgRNA sequences designed by CRISPR designer at http://crispr.mit.edu/ were ligated into LentiCRISPRv2 plasmid, which was then cotransfected with viral packaging plasmids (psPAX2 and pMD2G) into HEK293T cells. Forty‐eight hours after transfection, viral supernatant was harvested and then filtered through a 0.45‐μm strainer. HCT116/DLD1 cells were infected by viral supernatant. Selection was performed with puromycin (1 μg·mL^−1^) for 1 week. For LDHB‐re‐expressed cell lines, cDNA of the wild‐type (WT) LDHB and K293R and K293Q mutants were subcloned into pLenti‐CMV‐EGFP‐3Flag‐PGK‐Puro vector. These plasmids were cotransfected with viral plasmids (vsvg and gag) into HEK293T cells, respectively. Forty‐eight hours after transfection, viral supernatant was filtered through a 0.45‐μm strainer and infected into HCT116/DLD1 LDHB‐KO cells. Infected cells were screened by monoclonal cultivation to obtain stably re‐expressed cell lines verified by western blotting. The following guide sequences were used for CRISPR–Cas9 knockout: SIRT5 (sense 5ʹ‐CACCGGCTGGGAAATCAATCGACTT‐3ʹ, antisense 5ʹ‐AAACAAGTCGATTGATTTCCCAGCC‐3ʹ) and LDHB (sense 5ʹ‐CACCGACTACAGTGATCTTATTGTT‐3ʹ, antisense 5ʹ‐AAACAACAATAAGATCACTGTAGTC‐3ʹ).

### 2.3 RNA interference and cell transfection

Lipofectamine 2000 (Invitrogen, Waltham, MA, USA) was used in transient transfection according to the manufacturer's protocol. The sequences of siRNA oligos were as follows: SIRT5 (sense 5ʹCGUCCACACGAAACCAGAUUU‐3ʹ, antisense 5ʹ‐AAAUCUGGUUUCGUGUGGACG‐3ʹ), LDHB (sense 5ʹ‐GGAUAUACCAACUGGGCUATT‐3ʹ, antisense 5ʹ‐UAGCCCAGUUGGUAUAUCCTT‐3ʹ) and negative control: (sense 5ʹ‐UUCUCCGAACGUGUCACGUTT‐3ʹ, antisense 5ʹ‐ACGUGACACGUUCGGAGAATT‐3ʹ).

Vector pLV‐U6‐EGFP‐Puro expressing shRNA was used for constructing stable knockdown or control cell lines. The sequences of shRNA used in this study were as follows: SIRT5 (sense 5ʹ‐CCGGGGAGATCCATGGTAGCTTACTCGAGTAAGCTACCATGGATCTCCTTTTTG‐3ʹ, antisense 5ʹ‐AATTCAAAAAGGAGATCCATGGTAGCTTACTCGAGTAAGCTACCATGGATCTCC‐3ʹ), LDHB (sense 5ʹ‐CCGGGGATATACCAACTGGGCTACTCGAGTAGCCCAGTTGGTATATCCTTTTTG‐3ʹ, antisense 5ʹ‐AATTCAAAAAGGATATACCAACTGGGCTACTCGAGTAGCCCAGTTGGTATATCC‐3ʹ) and negative control (sense 5ʹ‐CCGGTTCTCCGAACGTGTCACGTTCTCGAGACGTGACACGTTCGGAGTTTTTG‐3ʹ, antisense 5ʹ‐ AATTCAAAAATTCTCCGAACGTGTCACGTTCTCGAGACGTGACACGTTCGGAG‐3ʹ).

### 2.4 RNA extraction and quantitative real‐time polymerase chain reaction

Total RNA was isolated using TRIzol reagent (Omega, Norcross, GA, USA) and transcribed to cDNA using a cDNA synthesis kit (Takara, Dalian, China) according to the manufacturer's protocol. Quantitative real‐time PCR was carried out by using SYBR Green PCR Master Mix (Takara, Shiga, Japan). The StepOnePlus Real‐Time PCR System (Applied Biosystems, Waltham, MA, USA) was used to detect the transcript levels of the genes. The gene‐specific primers were as follows: LDHB (sense 5ʹ‐ATGGCAACTCTAAAGGATCAGC‐3ʹ, antisense 5ʹ‐CCAACCCCAACAACTGAATCT‐3ʹ) and CB (sense 5ʹ‐AGATGTAGGCCGGGTGATCT‐3ʹ, antisense 5ʹ‐CCGCCCTGGATCATGAAGTC‐3ʹ).

### 2.5 Confocal immunofluorescence microscopy

HEK293T and HCT116 cells were grown in 24‐well plates on glass coverslips. Cells were harvested, washed with PBS and fixed using 4% paraformaldehyde for 10 min. After permeabilization with 0.25% Triton X‐100 for 10 min, the cells were washed with PBS and blocked with 1% bovine serum albumin (BSA)/PBS for 30 min, followed by incubation with primary antibodies overnight at 4 °C and subsequently with a secondary antibody at room temperature for 1 h. Images were acquired on a confocal laser‐scanning microscope (Zeiss, Oberkochen, Germany). The results were from two independent experiments, performed in triplicate.

### 2.6 Mass spectrometry

Cell lysate including Flag‐tagged vector/SIRT5 protein was incubated with 15 μL of anti‐Flag agarose (Sigma‐Aldrich) overnight at 4 °C. Then, the samples were subjected to electrophoresis followed by Coomassie Blue staining. Then, the gel was destained with 50% acetonitrile containing 50 mm NH_4_HCO_3_ and reduced with 100 mm NH_4_HCO_3_. Then, gel pieces were dehydrated with 100% acetonitrile for 5 min. After the liquid was removed, the gel pieces were rehydrated in dithiothreitol (10 mm) at 37 °C for 60 min. Gel pieces were again dehydrated in 100% acetonitrile. After the liquid was removed, gel pieces were rehydrated with iodoacetamide (55 mm). Samples were incubated for 45 min at room temperature (RT) in dark place. Gel pieces were washed with NH_4_HCO_3_ (50 mm) and dehydrated again with 100% acetonitrile. Finally, the pellet was resuspended using NH_4_HCO_3_ (50 mm) containing 10 ng·μL^−1^ trypsin and incubated on ice for 1 h. Excess liquid was removed and gel pieces were digested with trypsin at 37 °C overnight. Peptides were sequentially extracted with 50% acetonitrile/5% formic acid and 100% acetonitrile. The sample was then dried and further analysed by LC‐MS/MS. In LC‐MS/MS analysis, peptides were dissolved in 0.1% formic acid (solvent A). Solvent B was 100% acetonitrile containing 0.1% formic acid. Gradient separation setting: 0–1 min 3–7% solvent B; 1–46 min 7–22% solvent B; 46–55 min 22–35% solvent B; and 55–56 min 35–80% solvent B, then holding at 80% for 4 min. Flow rate is maintained at 300 nL·min^−1^. The peptides were injected into an NSI source for ionization and then analysed by Thermo Scientific VELOS PRO mass spectrometry (Waltham, MA, USA). Voltage: 2.0 kV. The peptide precursor and its secondary fragments were detected and analysed using high‐resolution Orbitrap. The *m*/*z* scan range was 350–1800 for full scan, and intact peptides were detected at a resolution of 60 000. The fragments were detected in the Orbitrap at a resolution of 17 500. The dynamic exclusion time of the tandem mass spectrometry scan was set to 15.0 s. Automatic gain control (AGC) was set at 5E4. The resulting MS/MS data were processed using Proteome Discoverer 2.0. Database: human identification (Thermo Scientific).

### 2.7 GST pull‐down

GST‐tagged SIRT5 and His‐tagged LDHB were expressed in BL21(DE3) cells (Sangon Biotech, Shanghai, China). GST‐tagged proteins were purified with Glutathione Sepharose 4B beads (GE Healthcare, Chicago, IL, USA) according to the manufacturer's instructions. His‐tagged proteins were prepared and purified using Ni‐affinity resins (GE Healthcare). Purified GST‐tagged SIRT5 protein was incubated with His‐tagged LDHB protein at 4 °C for 1 h. The beads were washed 5–10 times and boiled in SDS loading buffer. Then, samples were analysed by western blotting.

### 2.8 Immunoprecipitation

To analyse endogenous protein–protein interaction, whole lysates were incubated with antibody against LDHB or SIRT5 and 20 μL protein A/G agarose (Pierce, Waltham, MA, USA) overnight at 4 °C. For exogenous co‐IP assay, cell lysate containing Flag‐tagged SIRT5 or HA‐tagged LDHB was incubated with anti‐Flag (Sigma‐Aldrich) or anti‐HA agarose (Sigma‐Aldrich) overnight at 4 °C. Then, 5× SDS/PAGE sample loading buffer was added to the agarose and boiled for 10 min. The resulting samples were analysed by western blotting.

### 2.9 Western blotting assay

Cells were washed with cold PBS and lysed in the RIPA buffer containing protease inhibitors by incubating for 30 min on ice, followed by centrifugation at 15 000 ***g*** for 30 min. Samples were boiled and then loaded on 10% or 15% SDS/PAGE, separated by electrophoresis and transferred to PVDF membranes, which were blocked and then incubated with the secondary antibodies for 1 h at room temperature. The immunoreactive bands were visualized by an ECL Plus system (Tanon, Shanghai, China).

### 2.10 Autophagic flux assay

An autophagic flux assay was performed using an mRFP‐GFP‐LC3 adenoviral vector‐encoding construct (HanBio Technology, Shanghai, China) to monitor autophagosome maturation, which was used according to the manufacturer's instructions (Zhou *et al*., 2012). The images were visualized using a confocal laser‐scanning microscope (Zeiss).

### 2.11 In vitro deacetylation

Acetylated HA‐LDHB protein was immunopurified using an anti‐HA antibody from TSA‐treated sh*SIRT5* HEK293T cells and then incubated with purified SIRT5 protein with or without 100 μm NAD^+^ or 5 mm nicotinamide, as indicated in the deacetylation reaction buffer (50 mm Tris/HCl, 137 mm NaCl, 2.7 mm KCl, 1 mm MgCl_2_, 1 mg·mL^−1^ BSA and 200 nm TSA, pH 8.0) for 1 h at 37 °C. After that, the samples were analysed by western blotting.

### 2.12 LDHB/LDHA activity assay

HA‐tagged LDHB/LDHA protein was immunopurified from transfected cells, and LDHB/LDHA activity was determined using an LDH activity assay kit according to the manufacturer's instructions (Njjcbio, Nanjing, China).

### 2.13 Immunohistochemistry

CRC samples were obtained from surgical patients who provided signed informed consent at Renji Hospital, Shanghai, China. The experiment was approved by the Ethics Committee of Renji Hospital. Subcutaneous tumour tissues of mice fixed in 4% paraformaldehyde were dehydrated, embedded in paraffin and cut into 4‐μm sections. Human colorectal tumour tissue samples or subcutaneous mouse tumour tissues were dewaxed, hydrated and washed. Antigens were retrieved with 10 mM sodium citrate buffer, and then, the slides were treated with 2% H_2_O_2_ in methanol to block endogenous peroxide, after which primary antibody was added and incubated at RT for 2 h. HRP‐conjugated goat anti‐rabbit IgG (Cell Signaling Technology) and DAB [3,30‐diaminobenzidine solution (DAKO, Copenhagen, Denmark)] were used, and counterstaining was performed with haematoxylin. The signal intensity of IHC was independently evaluated by two researchers without prior knowledge about the patients and samples. The signal intensity was divided into 0 = negative, 1 = weak, 2 = moderate and 3 = strong. The staining frequency was categorized as follows: 0 = no staining, 1 < 25%, 2 = 25–50% and 3 > 50%. The final scores for LDHB‐Ac‐K329 in those colorectal tissues were on a scale of 0–9, in which a score ≤ 3 was defined as representing low expression and a score of > 3 as representing high expression.

### 2.14 Lysosomal pH measurement

Lysosomes were loaded overnight with 0.5 mg·mL^−1^ of pH‐sensitive FITC/dextran (Sigma) prepared in DMEM. FITC/dextran was removed and washed in HBSS, followed by 2 h of incubation in HBSS with the indicated treatments. The emitted fluorescence was recorded using Axiovision software on a microscope equipped with an ApoTome module for structured illumination (Zeiss). The pH values were derived from the linear standard curve, which was obtained with the addition of 20 μm monensin (Sigma) and 10 μm nigericin (Invitrogen) in HEPES‐buffered HBSS adjusted to different pH values between 3.5 and 7.5. Fluorescence intensities of six images per condition were transformed into pH values using the standard curve.

### 2.15 XF24 extracellular flux analysis

We used an XF24 extracellular flux analyser to evaluate the oxygen consumption rate (OCR). In total, 1 × 10^4^ cells were plated in XF24 cell culture plates (Seahorse Bioscience, Billerica, MA, USA) and incubated at 37 °C overnight. Then, the cells were incubated in bicarbonate‐free DMEM medium in a 37 °C non‐CO_2_ incubator for 1 h, and the XF assay was performed at the designated time points. The values are presented as the means ± SE of the mean.

### 2.16 ATP and lactate assay

ATP Colorimetric Assay Kit (Biovision, Milpitas, CA, USA) was used to determine ATP production according to the manufacturer's instructions. Lactate levels in culture medium and in cells were measured according to the instructions of the Lactate Assay Kit (Njjcbio). These readouts were normalized by cell numbers.

### 2.17 Cell proliferation and colony‐formation assay

For all cell proliferation experiments, 5.0 × 10^4^ cells per well were seeded onto a 6‐well plate. Cells were collected every 24 h, and live cells were counted using a haemocytometer after trypan blue exclusion. For the plate colony‐formation assay, 200 cells per well were seeded onto a 6‐well plate. For the soft‐agar colony‐formation assay, 100 cells were plated onto a 24‐well plate. Cells were suspended in culture medium containing 0.4% agarose (Sigma) and placed on top of solidified 0.6% agarose in DMEM. The medium was changed regularly. After culture for 14 days, colonies were stained with crystal violet and counted.

### 2.18 Xenograft tumour studies

All *in vivo* experiments were performed in accordance with the guidelines provided by the Animal Ethics Committee of Renji Hospital. Five‐week‐old male BALB/c nude mice (Shanghai Laboratory Animal Center, Shanghai, China) were injected subcutaneously with LDHB‐KO HCT116/DLD1 cells (5 × 10^6^ cells) stably expressing EGFP‐3Flag‐LDHB^WT/K329R/K329Q^. All mice were killed at 21 days after injection, and tumour tissues were collected and weighed. Five‐week‐old male BALB/c nude mice (Shanghai Laboratory Animal Center) received subcutaneous injections of 5 × 10^6^ LDHB‐KO HCT116 cells that were stably transfected with EGFP‐3Flag‐LDHB^WT^ or EGFP‐3Flag‐LDHB^K329Q^ in 100 μL of saline at the right dorsal flank. Fourteen days after tumour implantation, the mice were randomly assigned to a treatment group receiving GW5074 (5 mg·kg^−1^) or a control group receiving saline three times per week intraperitoneally for 21 days. At the end of the experiment, animals were sacrificed, and tumour tissues were collected for weighing and immunohistochemical analysis.

### 2.19 Statistics

All data are presented as the means ± SEM and were statistically analysed using GraphPad Prism 6 (GraphPad Software, La Jolla, CA, USA) or SPSS 17 software (SPSS, Dallas, TX, USA). A two‐tailed unpaired *t*‐test or ANOVA was used to analyse the difference between two or more groups. The chi‐square test was applied for rate comparisons. The overall survival curves were calculated using the Kaplan–Meier method and compared by log‐rank test. *P*‐values < 0.05 were considered as statistically significant.

## 3 Results

### 3.1 SIRT5 interacts with LDHB

**Figure 1:**
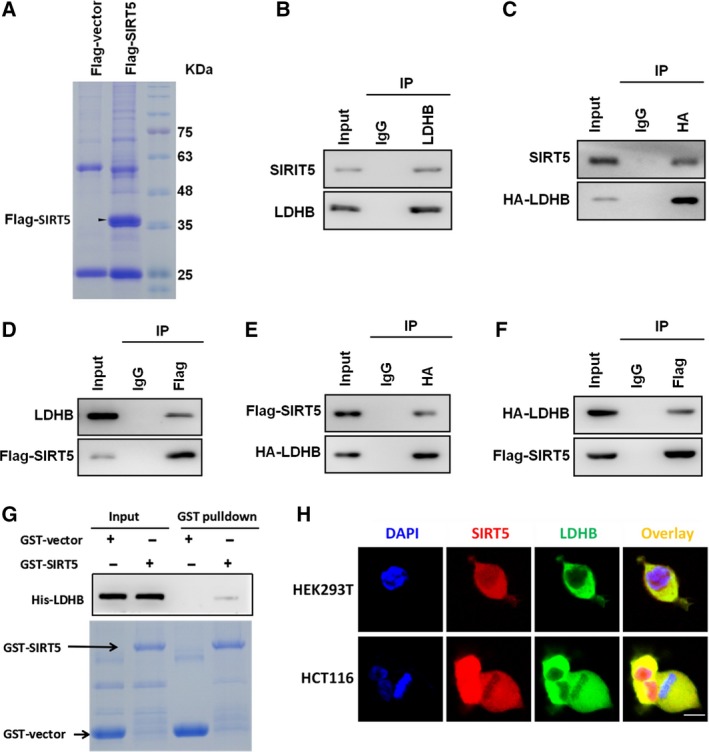
SIRT5 interacts with LDHB. (A) HEK293T cells were with transfected with Flag‐tagged SIRT5. Then, Flag‐tagged SIRT5 protein was immunoprecipitated from HEK293T cells lysates, followed by electrophoresis and Coomassie Blue staining. (B) Lysates of HEK293T were immunoprecipitated with the antibody against LDHB (IgG as control). Immunoprecipitates and whole‐cell extracts were subsequently separated by SDS/PAGE, followed by western blotting with an anti‐LDHB or SIRT5 antibody. (C) HEK293T cells were transfected with HA‐LDHB for 48 h. Total protein extracts were immunoprecipitated with anti‐HA antibody (IgG as a control) and separated by SDS/PAGE, followed by western blotting with an anti‐SIRT5 or HA antibody. (D) HEK293T cells were transfected with Flag‐SIRT5 for 48 h. Total protein extracts were immunoprecipitated with anti‐Flag antibody (IgG as a control) and separated by SDS/PAGE, followed by western blotting with an anti‐LDHB or anti‐Flag antibody. (E, F) HEK293T cells were cotransfected with HA‐LDHB and Flag‐SIRT5 for 48 h. Total protein extracts were immunoprecipitated with anti‐HA or anti‐Flag antibody (IgG as a control) and separated by SDS/PAGE, followed by western blotting with an anti‐HA or anti‐Flag antibody. (G) Recombinant GST–SIRT5 (or GST–vector as a control) was incubated with purified His‐LDHB protein for 3 h. Bound proteins were eluted and analysed with SDS/PAGE and Coomassie Blue staining. Anti‐His antibody was used to detect LDHB in eluates. (H) The colocalization (yellow) of endogenous SIRT5 (red) and LDHB (green) in HEK293T and HCT116 cells was analysed using an immunofluorescence and confocal laser‐scanning microscopy. Scale bar, 10 μm.

Based on the importance of SIRT5 in tumour progression, we asked which regulatory signalling factors were directly regulated by SIRT5. Most previous studies have mainly focused on the PTM functions of SIRT5 in glycolysis, ammonia metabolism, FAO and oxidative stress (Colak *et al*., 2015; Lin *et al*., 2013; Nakagawa *et al*., 2009; Nishida *et al*., 2015; Wang *et al*., 2018; Zhou *et al*., 2016). To search for novel interacting partners of SIRT5, we overexpressed C‐terminal Flag‐tagged SIRT5 protein in HEK293T cells (Fig. 1A) and then performed mass spectrometry to identify the associated proteins. We identified LDHB as a novel interacting partner protein of SIRT5. The SIRT5–LDHB interaction was then confirmed by direct endogenous and exogenous endogenous co‐immunoprecipitation (co‐IP) in HEK293T cells (Fig. 1B, endogenous interaction; Fig. 1C–F, exogenous interaction). In the GST pull‐down assay, we further found that GST‐tagged SIRT5 interacted with recombinant LDHB protein (Fig. 1G). Immunofluorescence staining showed that SIRT5 and LDHB colocalized in the cytoplasm in HEK293T and HCT116 cells (Fig. 1H). Therefore, LDHB functions as a physiological interacting partner of SIRT5.

### 3.2 SIRT5 deacetylates LDHB at lysine‐329

**Figure 2:**
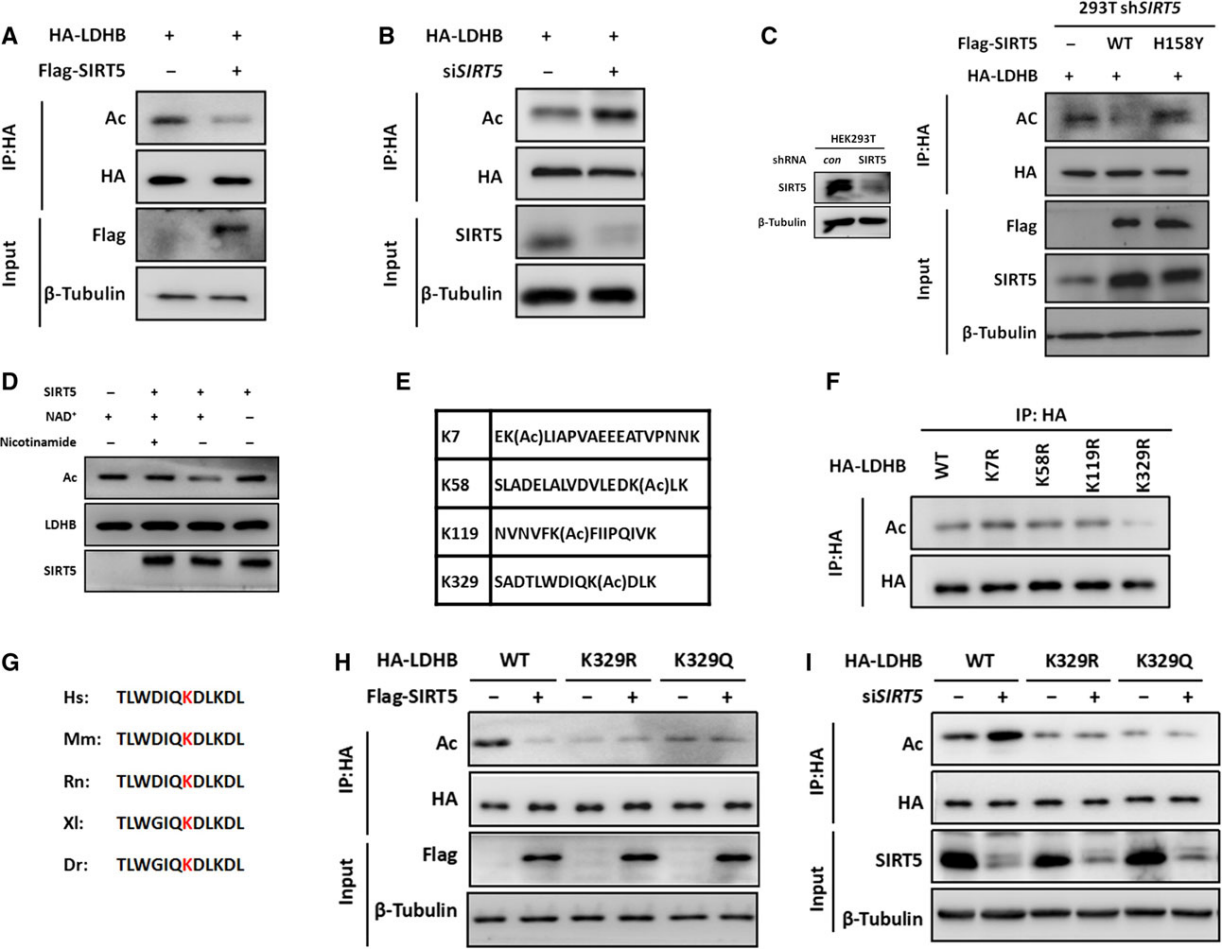
SIRT5 deacetylates LDHB at lysine‐329. (A) HCT116 cells were transfected with HA‐LDHB and Flag‐SIRT5 (or empty vector as a control) for 48 h. Cell lysates were immunoprecipitated with HA‐LDHB affinity gel. LDHB acetylation and protein levels were analysed by immunoblotting using the indicated antibodies. (B) HCT116 cells were transfected with HA‐LDHB and siRNA against SIRT5 (or negative control siRNA) for 48 h. Proteins were immunoprecipitated and LDHB acetylation was determined by western blotting. (C) Left: SIRT5 was stably knocked down in HEK293T cells and detected by immunoblotting. Right: HEK293T sh*SIRT5* cells were cotransfected with HA‐LDHB and Flag‐vector/SIRT5 wild‐type (WT)/SIRT5 inactive mutant (H158Y). Proteins were immunoprecipitated and LDHB acetylation was determined by western blotting. (D) In vitro deacetylation of LDHB by SIRT5 is shown. Affinity‐purified acetylated HA‐LDHB proteins were incubated with or without recombinant SIRT5 protein in buffers containing the indicated chemicals as described in the Materials and methods section. (E) Identification of acetylated LDHB peptides by mass spectrometry. (F) Analysis of acetylation of individual LDHB mutants. HA‐tagged wild‐type and mutant LDHB proteins were expressed in HCT116 cells and purified by immunoprecipitation. LDHB acetylation was determined by western blotting. (G) Alignment of protein sequences surrounding K329 of LDHB from different organisms. *Hs: Homo sapiens, human; Mm: Mus musculus, mouse; Rn: Rattus norvegicus, Norway rat; Xl: Xenopus laevis, frog; Dr: Danio rerio, zebrafish*. (H) HCT116 cells were cotransfected with Flag‐SIRT5 (or empty vector as a control) and HA‐LDHB^WT^, HA‐LDHB^K^ ^329R^ or HA‐LDHB^K^ ^329Q^ for 48 h. Acetylation level of immunoprecipitated HA‐LDHB was measured by direct western blotting using the acetyl‐LDHB (K329) antibody (K329‐Ac). (I) HCT116 cells were cotransfected with siRNA‐SIRT5 (or siRNA‐NC as a control) and HA‐LDHB^WT^, HA‐LDHB^K^ ^329R^ or HA‐LDHB^K^ ^329Q^ for 48 h. Proteins were immunoprecipitated and acetylation of K329 was determined by western blotting.

Given that SIRT5 has been demonstrated to regulate metabolic enzymes by direct PTM (Yang *et al*., 2015), we asked whether this interaction could affect the lysine acetylation of LDHB. We found that SIRT5 overexpression decreased LDHB acetylation in HCT116 cells, while the succinylation, malonylation or glutarylation of LDHB remained largely unaffected (Fig. 2A and Fig. S1). Supporting this observation, SIRT5 knockdown using specific siRNA increased LDHB acetylation (Fig. 2B). Furthermore, we stably knocked down SIRT5 in HEK293T cells (Fig. 2C, left) and found that only SIRT5‐WT, but not enzymatically inactive SIRT5‐H158Y, deacetylated LDHB (Fig. 2C, right). Finally, *in vitro* deacetylation assays confirmed that affinity‐purified acetyl‐HA‐LDHB protein can be directly deacetylated by SIRT5 protein in a NAD^+^‐dependent manner (Fig. 2D). Collectively, these data indicated that LDHB is a deacetylation substrate of SIRT5 in cells and *in vitro*.

Four putative acetylation sites were identified in LDHB by mass spectrometry (Fig. 2E) (Choudhary *et al*., 2009). We then mutated each of the four putative acetylation sites individually to arginine (R). The mutation of K329, but not other lysine residues, to arginine resulted in a significant reduction in LDHB acetylation (Fig. 2F). This result indicates that K329, which is evolutionarily conserved from *Danio rerio* to mammals (Fig. 2G), is a major acetylation site in LDHB. Afterwards, we generated an antibody specifically recognizing the K329‐acetylated LDHB. We found that SIRT5 had no significant effect on the acetylation of LDHB^K329R^ and LDHB^K329Q^ [a mimic LDHB acetylation mutant created by substituting K329 with glutamine (Q)] mutants (Fig. 2H,I). Thus, we demonstrated that LDHB was deacetylated by SIRT5 at K329.

### 3.3 SIRT5‐mediated deacetylation increases LDHB activity

**Figure 3:**
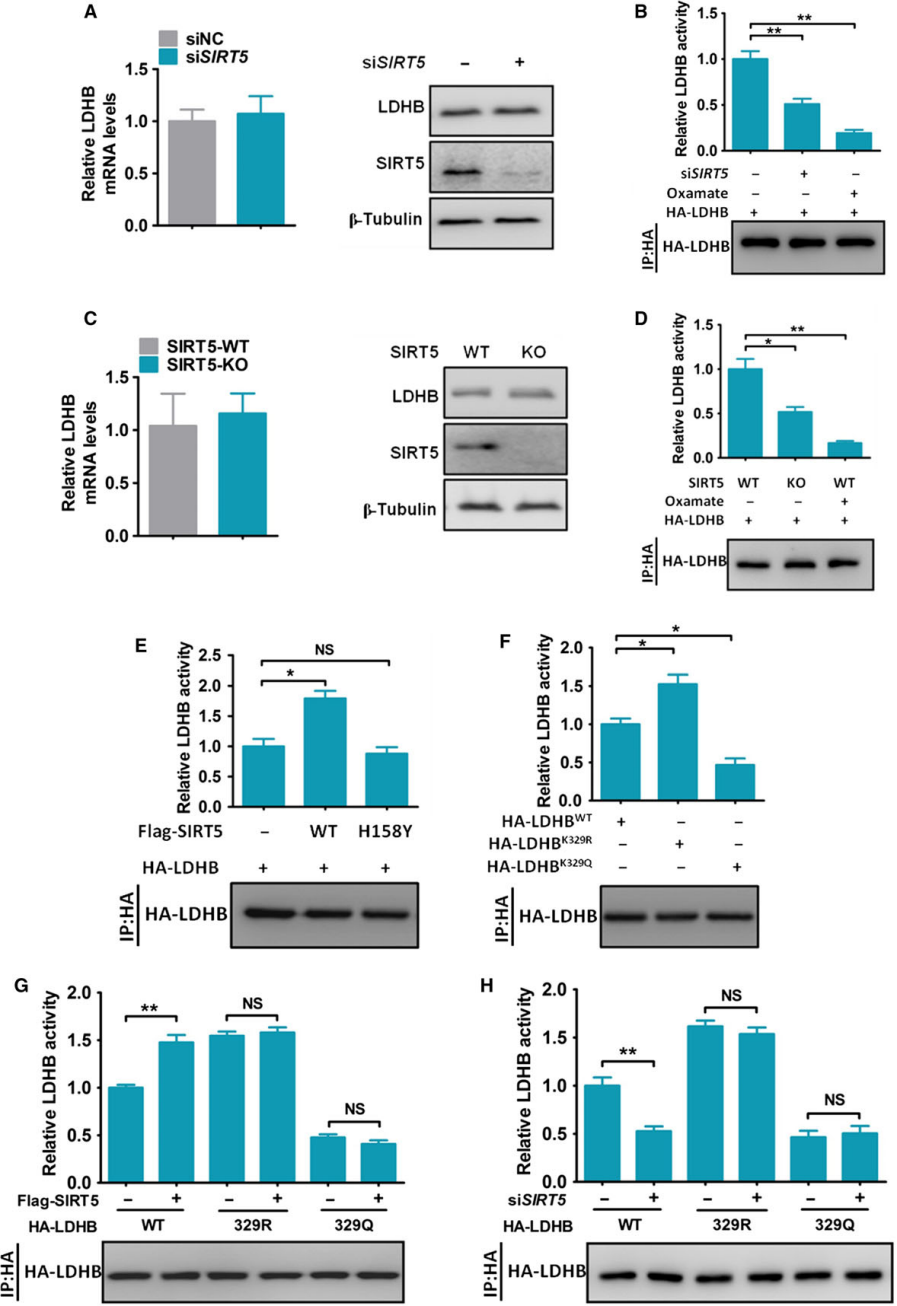
SIRT5‐mediated deacetylation increases LDHB activity. (A) HCT116 cells were transfected with siRNA‐SIRT5 (or siRNA‐NC as a control) for 48 h. The level of LDHB mRNA was analysed with qPCR. LDHB protein was analysed with western blotting. β‐Tubulin was used as a loading control for western blotting. Error bars represent means ± SEM (*n* = 3). (B) HCT116 cells were transfected with HA‐LDHB and siRNA against SIRT5 or negative control siRNA for 48 h. In addition, control cells were treated with the LDHB inhibitor oxamate (10 mm) for the last 24 h. HA‐LDHB was immunoprecipitated, and LDHB activity was assayed. LDHB activity was normalized against protein levels. Error bars represent means ± SEM (*n* = 3). (C) LDHB mRNA and protein levels were analysed in SIRT5 WT HCT116 cells or SIRT5 KO HCT116 cells. Error bars represent means ± SEM (*n* = 3). (D) SIRT5 WT/KO HCT116 cells were transfected with HA‐LDHB for 48 h. In addition, WT cells were treated with the LDHB inhibitor oxamate (10 mm) for the last 24 h. LDHB was immunoprecipitated, and LDHB activity was assayed. LDHB activity was normalized against protein levels. Error bars represent means ± SEM (*n* = 3). (E) HEK293T sh*SIRT5* cells were cotransfected with HA‐LDHB and vector/SIRT5 wild‐type (WT)/SIRT5 inactive mutant (H158Y). LDHB was immunoprecipitated, and LDHB activity was assayed. LDHB activity was normalized against the protein levels. Error bars represent means ± SEM (*n* = 3). (F) HA‐tagged wild‐type and mutant LDHB proteins were expressed in HCT116 cells and purified by immunoprecipitation. The enzyme activity was measured and normalized against the protein level. Error bars represent means ± SEM (*n* = 3). (G) HCT116 cells were cotransfected with Flag‐SIRT5 (or empty vector as control) and HA‐LDHB^WT^, HA‐LDHB^K^ ^329R^ or HA‐LDHB^K^ ^329Q^ for 48 h. LDHB was immunoprecipitated, and LDHB activity was assayed. LDHB activity was normalized against the protein levels. Error bars represent means ± SEM (*n* = 3). (H) HCT116 cells were cotransfected with siRNA‐SIRT5 (or siRNA‐NC as a control) and HA‐LDHB^WT^, HA‐LDHB^K^ ^329R^ or HA‐LDHB^K^ ^329Q^ for 48 h. LDHB was immunoprecipitated, and LDHB activity was assayed. LDHB activity was normalized against the protein levels. Error bars represent means ± SEM (*n* = 3). *P < 0.05, **P < 0.01. *P*‐values were based on Student's *t*‐test.

To determine whether the SIRT5‐mediated deacetylation modification regulated LDHB expression or function, we initially knocked down SIRT5 in CRC cells using specific siRNA. SIRT5 knockdown had no effects on the mRNA or protein levels of LDHB (Fig. 3A) but resulted in a decrease in LDHB activity in HCT116 and DLD1 cells (Fig. 3B and Fig. S2A). SIRT5 KO had similar effects on LDHB activity in HCT116 cells (Fig. 3C,D). In contrast, we verified that si*SIRT5* or SIRT5 KO did not alter LDHA activity in CRC cells (Fig. S2B,C). Furthermore, re‐expression of wild‐type SIRT5, but not the inactive H158Y mutant, increased LDHB enzyme activity in HEK293T sh*SIRT5* cells (Fig. 3E).

To test the effect of K329 acetylation, the activities of LDHB^K329R^ and LDHB^K329Q^ mutants were compared with that of wild‐type LDHB. We found that LDHB^K329Q^ displayed only 50% of the wild‐type activity, whereas the LDHB^K329R^ mutation produced an opposite effect on LDHB activity in HCT116 and DLD1 cells (Fig. 3F and Fig. S2D). SIRT5 regulated the activity of the LDHB^WT^, but not LDHB^K329R^ and LDHB^K329Q^ mutants (Fig. 3G,H), indicating that SIRT5 stimulated LDHB activity mostly via deacetylation of K329. These data demonstrated that LDHB activity was regulated by SIRT5 via deacetylation modification at K329.

### 3.4 SIRT5 promotes autophagy through LDHB deacetylation

**Figure 4:**
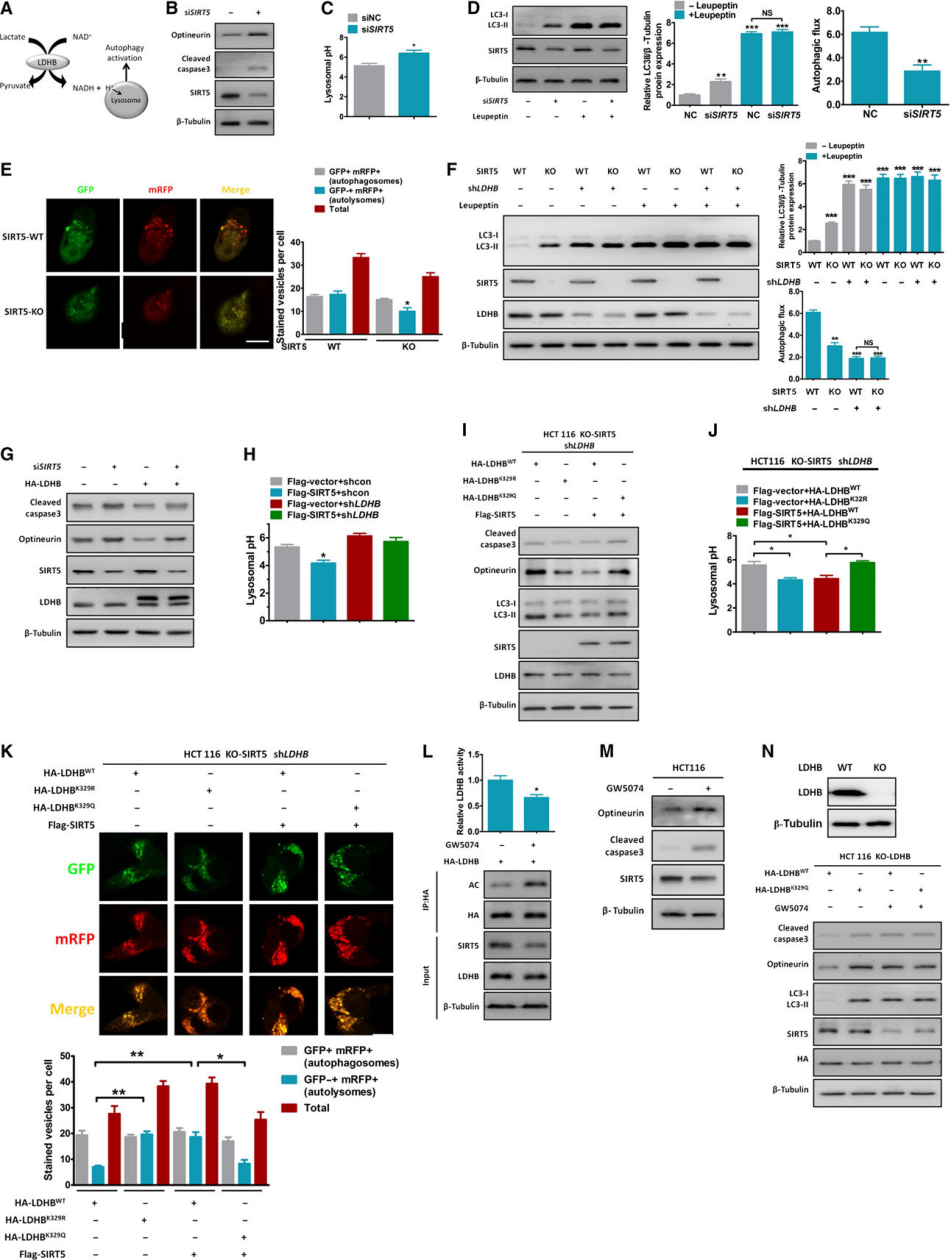
SIRT5 promotes autophagy through LDHB deacetylation. (A) A schematic of the LDHB‐mediated autophagy pathway. (B–C) HCT116 cells were transfected with siRNA‐SIRT5 (or siRNA‐NC as a control) for 48 h. Protein levels of optineurin and cleaved caspase‐3 were analysed with western blotting (B), and lysosomal pH was measured with FITC/dextran (C). Error bars represent means ± SEM (*n* = 3). (D) HCT116 cells were transfected with siRNA‐SIRT5 (or siRNA‐NC as a control) for 48 h, followed by 10 h of leupeptin (150 mm) treatment. Protein levels of SIRT5 and LC3‐II were analysed with western blotting. Autophagic flux was determined by the difference in LC3‐II expression between conditions with and without leupeptin. Error bars represent means ± SEM (*n* = 3). (E) SIRT5 WT or KO HCT116 cells were infected with virus suspension containing mRFP‐EGFP‐LC3 reporter for 2 h under normal conditions. The abundance of autophagosomes and autolysosomes was measured using a confocal microscope. Error bars represent means ± SEM (*n* = 3). Scale bar, 10 μm. (F) SIRT5‐WT or SIRT5‐KO HCT116 cells stably expressing shRNA‐LDHB (sh*LDHB*) or shRNA‐NC (shNC) were or were not treated with leupeptin (150 mm) for 10 h. The LDHB, SIRT5, LC3‐I and LC3‐II protein abundance is shown. Autophagic flux was calculated as the difference in LC3‐II expression between conditions with and without leupeptin. Error bars represent means ± SEM (*n* = 3). (G) HCT116 cells were transfected with si*SIRT5* or siNC as control, and HA‐LDHB or HA‐vector as control for 48 h. Optineurin and cleaved caspase‐3 protein levels are shown. (H) sh*LDHB* or shNC HCT116 cells were transfected with Flag‐SIRT5 or Flag‐vector as controls for 48 h. Lysosomal pH measured with FITC/dextran. Error bars represent means ± SEM (*n* = 3). (I–K) SIRT5 KO and sh*LDHB* HCT116 cells were transfected with HA‐LDHB^WT^, HA‐LDHB^K^ ^329R^, HA‐LDHB^WT^+Flag‐SIRT5 or HA‐LDHB^K^ ^329Q^+Flag‐SIRT5. (I) Autophagic and apoptotic protein levels are shown. (J) Lysosomal pH was measured with FITC/dextran. Error bars represent means ± SEM (*n* = 3). (K) Abundance of autophagosomes and of autolysosomes. Scale bar, 10 μm. Error bars represent means ± SEM (*n* = 3). (L) HCT116 cells were transiently transfected with HA‐LDHB^WT^ for 48 h and treated or not treated with 100 μm GW5074 for the last 24 h, followed by immunoprecipitation, LDHB enzyme activity assay and LDHB acetylation assay by western blotting. Error bars represent means ± SEM (*n* = 3). (M) Autophagic and apoptotic protein abundance in HCT116 cells treated or not treated with 100 μm GW5074 for 24 h. (N) LDHB was knocked out in HCT116 cells, and LDHB and β‐tubulin levels were determined by western analysis using anti‐LDHB and β‐tubulin antibodies. LDHB‐KO HCT116 cells were transfected with HA‐LDHB^WT^ or HA‐LDHB^K^ ^329Q^ for 48 h and treated or not treated with 100 μm GW5074 for the last 24 h. Autophagic and apoptotic protein abundance is shown. *P < 0.05, **P < 0.01, ***P <* *0.001. *P*‐values were based on Student's *t*‐test.

A previous study showed LDHB activity to be necessary for basal autophagy and cancer cell proliferation since it directly controls lysosomal acidification (Fig. 4A; Brisson *et al*., 2016). SIRT5‐mediated deacetylation of LDHB increased LDHB activity in our study. Thus, we examined possible autophagy regulation by SIRT5. Autophagic impairment often results in accumulation of the autophagic substrate optineurin (Korac *et al*., 2013). Silencing SIRT5 increased optineurin accumulation and apoptosis (cleaved caspase‐3) levels (Fig. 4B) and decreased lysosome acidification in HCT116 cells (Fig. 4C). To demonstrate that SIRT5 controls autophagic flux in cancer cells, we determined the LC3‐II protein level, a marker of the autophagic flux (Rubinsztein *et al*., 2009). SIRT5 knockdown induced leupeptin‐sensitive LC3‐II protein accumulation in HCT116 cells, which represents a potent inhibition of the autophagic flux (6.17–2.87) (Fig. 4D). Furthermore, we transiently transfected a tandem mRFP‐EGFP‐LC3 reporter to estimate the autophagic flux by measuring the abundance of mature autolysosomes (Kimura *et al*., 2007) and found that SIRT5 KO decreased the number of mature autolysosomes in HCT116 cells (Fig. 4E).

To examine whether the SIRT5‐mediated regulation of autophagy was LDHB‐dependent, LDHB was stably knocked down in SIRT5 WT/KO HCT116 cells by sh*LDHB*. Knocking out SIRT5 caused LC3‐II protein accumulation and autophagic flux inhibition with no additive effect from sh*LDHB* (Fig. 4F). LDHB overexpression abolished the si*SIRT5*‐mediated autophagy inhibition and pro‐apoptotic effect (Fig. 4G). Moreover, SIRT5 overexpression significantly promoted lysosomal acidification (Fig. 4H). However, LDHB knockdown by specific siRNA almost completely compromised SIRT5‐induced lysosomal acidification in the HCT116 cells (Fig. 4H). Thus, our results show that the effect of SIRT5 on autophagy is largely LDHB‐dependent.

Furthermore, SIRT5 KO and sh*LDHB* HCT116 cells were subsequently transfected with HA‐LDHB^WT^, HA‐LDHB^K329R^, HA‐LDHB^WT^+Flag‐SIRT5 or HA‐LDHB^K329Q^+Flag‐SIRT5. Cells expressing the deacetylated LDHB protein (HA‐LDHB^K329R^ or HA‐LDHB^WT^+Flag‐SIRT5 group) exhibited a decrease in apoptosis and an accumulation of autophagic substrate (Fig. 4I), and promotion in lysosome acidification (Fig. 4J), and autolysosome formation (Fig. 4K). Similar effects were observed in DLD1 cells (Fig. S3A–C). GW5074 is an inhibitor of SIRT5 with pharmacologically suitable properties (Suenkel *et al*., 2012). We observed that GW5074 treatment reduced SIRT5 protein levels and increased LDHB‐K329 acetylation (Fig. 4L), followed by an inhibition of autophagy and an induction of apoptosis (Fig. 4M). When SIRT5 was deregulated by GW5074, ectopic expression of LDHB^WT^ and LDHB^K329Q^ in LDHB‐KO HCT116 cells resulted in a comparable level of autophagy and apoptosis (Fig. 4N). These results suggest, for the first time, that the acetylation status of LDHB, as directed by SIRT5, determines its enzymatic activity and regulates autophagy as well as apoptosis in CRC cells.

### 3.5 LDHB deacetylation by SIRT5 promotes cell respiration, proliferation and tumour growth

As LDHB is a glucose metabolic enzyme oxidating lactate to pyruvate, we tested the effect of LDHB‐K329 acetylation on metabolism. HCT116 LDHB‐KO cells were infected with EGFP‐3Flag‐LDHB^WT/K329R/K329Q^ to stably overexpress these genes (Fig. S4A). We found that LDHB K329 deacetylation significantly decreased intracellular and extracellular lactate (LDHB reaction substrate) levels in HCT116 cells (Fig. S4B). We also observed that oxygen consumption rate (OCR) reflecting cell respiration and ATP production were increased in cells transfected with the LDHB K329R deacetylated mutant and decreased with the LDHB K329Q‐acetylated mutant (Fig. S4C,D). Together, these data show that LDHB K329 deacetylation promotes lactate‐fuelled cell respiration.

**Figure 5:**
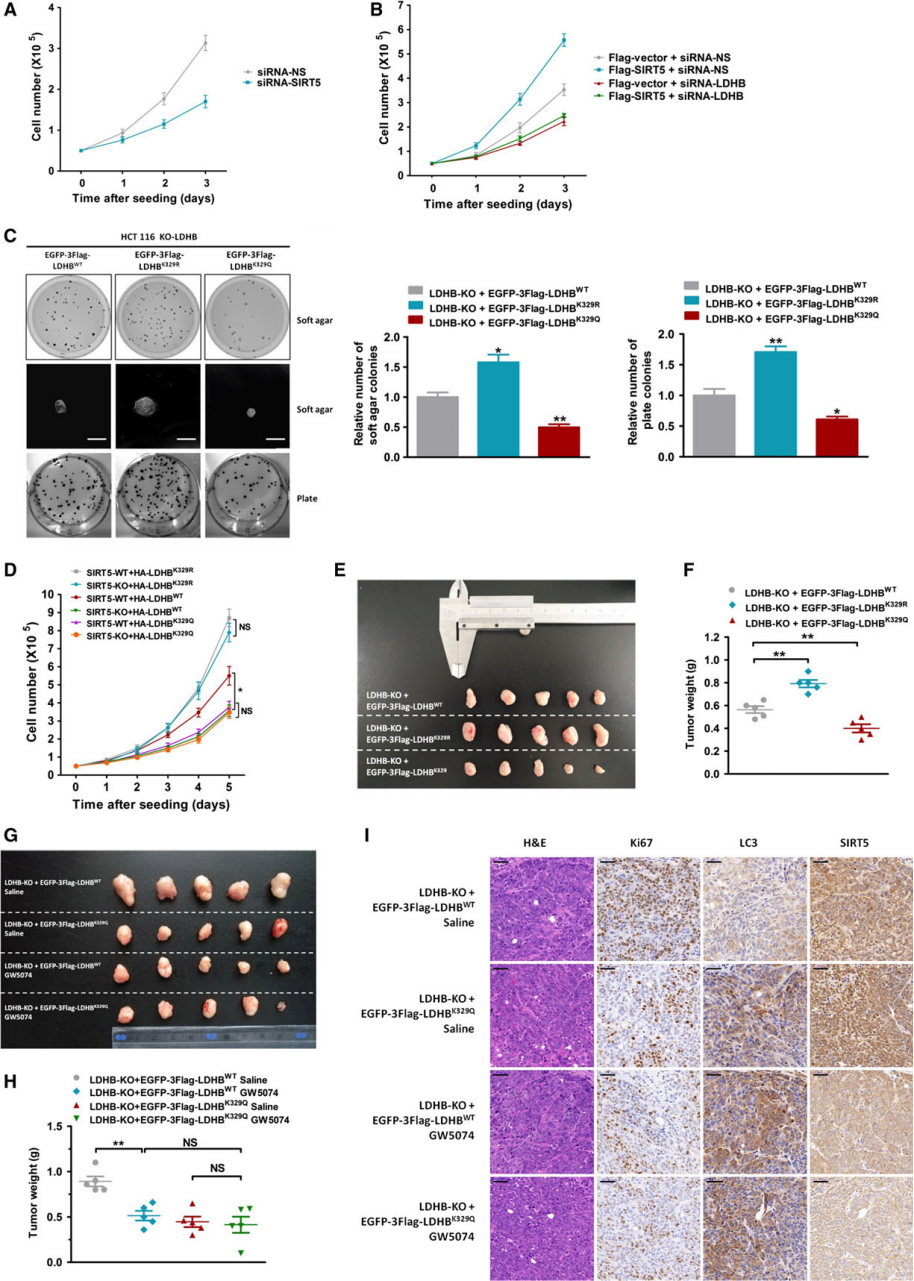
Acetylation of LDHB at K329 regulates tumour cell growth *in vivo* and *in vitro*. (A) After transfection with siRNA‐SIRT5 (or siRNA‐NS as a control), the numbers of HCT116 cells were counted every 24 h. Error bars represent means ± SEM (*n* = 3). (B) After transfection with siRNA‐LDHB (or siRNA‐NS as a control) and Flag‐SIRT5 (or empty vector as a control), the numbers of HCT116 cells were counted every 24 h. Error bars represent means ± SEM (*n* = 3). (C). Soft‐agar colony‐formation assays (C, top and middle) and plate colony‐formation assays (C, bottom) of LDHB‐KO HCT116 cells stably expressing EGFP‐3Flag‐LDHB^WT^ ^/K329R/K329Q^. Error bars represent means ± SEM (*n* = 3). (D) Numbers of SIRT5 WT or SIRT5 KO HCT116 cells transfected with HA‐LDHB^WT^/HA‐LDHB^K^ ^329R^/HA‐LDHB^K^ ^329Q^ every 24 h after transfection. Error bars represent means ± SEM (*n* = 3). (E) A total of 5 × 10^6^ LDHB‐KO HCT116 cells stably expressing EGFP‐3Flag‐LDHB^WT^ ^/K329R/K329Q^ were injected subcutaneously into the right dorsal flank of nude mice. Image of tumours isolated from nude mice (*n* = 5 for each group). (F) The weight of tumours when mice were sacrificed. (G–I) A total of 5 × 10^6^ LDHB‐KO HCT116 cells stably expressing EGFP‐3Flag‐LDHB^WT^ ^/K329Q^ were injected subcutaneously into the right dorsal flank of nude mice. Fourteen days after tumour implantation, mice were randomly assigned to a treatment group receiving GW5074 (5 mg·kg^−1^) or saline as a control group three times per week intraperitoneally for 35 days (*n* = 5 for each group). (G) Image of tumours isolated from nude mice. (H) The weight of tumours when mice were sacrificed. (I) Haematoxylin and eosin (H&E) staining and immunohistochemical detection of Ki67, LC3 and SIRT5. Scale bar, 50 μm. *P < 0.05, **P < 0.01. *P*‐values were based on Student's *t*‐test.

It is well known that increased autophagic flux and lactate‐fuelled cell respiration are related to oxidative cancer cell proliferation (Brisson *et al*., 2016; Sonveaux *et al*., 2008). We further assessed the contribution of the SIRT5/LDHB axis to these events. As shown in Fig. 5A, SIRT5 knockdown by specific siRNA significantly decreased cell number in HCT116 cells. Conversely, overexpression of SIRT5 in HCT116 cells increased cell number (Fig. 5B). Furthermore, SIRT5‐induced cell proliferation effect was completely compromised by LDHB knockdown (Fig. 5B), suggesting that the SIRT5‐mediated cell proliferation advantage in HCT116 cells was LDHB‐dependent. To further examine the role of LDHB K329 deacetylation by SIRT5 in cell proliferation, we performed plate and soft‐agar colony‐formation assay. Compared with the LDHB^WT^‐transfected cells, we observed significantly more colonies in the nonacetylatable LDHB^K329R^‐transfected cells while less colonies in the LDHB^K329Q^‐transfected cells (Fig. 5C). SIRT5 KO caused a significant decrease of cell number in HCT116 cells transfected with LDHB^WT^, whereas the number of cells expressing LDHB ^K329R^ or LDHB ^K329Q^ was comparable to that in the control group, regardless of the expression of SIRT5 (Fig. 5D). After the cell proliferation advantage afforded by SIRT5‐mediated LDHB deacetylation to cancer cells in vitro was verified, we performed xenograft studies *in vivo*. In xenograft mouse models, LDHB‐KO HCT116 or DLD1 cells with stable expression of EGFP‐3Flag‐LDHB^WT/K329R/K329Q^ were inoculated into nude mice. As shown in Fig. 5E,F and Fig. S5A‐C, replacement of endogenous LDHBWT by LDHBK329Q reduced tumour growth and replacement of endogenous LDHBWT by LDHBK329R promotes tumour growth. In case of GW5074 treatment, ectopic expression of either LDHB WT or K329Q resulted in comparable tumour volumes and weights (Fig. 5G,H). Consistent with our previous *in vitro* results, IHC analysis of tumours also revealed that GW5074 reduced SIRT5 protein level and resulted in a significant downregulation of autophagic flux and cell proliferation (Fig. 5I). Collectively, these in vivo and in vitro results indicated the K329 deacetylation of LDHB by SIRT5 promoted cancer cell proliferation and tumour growth.

### 3.6 Decreased LDHB‐K329 acetylation in human CRC samples and correlation with poor prognosis

**Figure 6:**
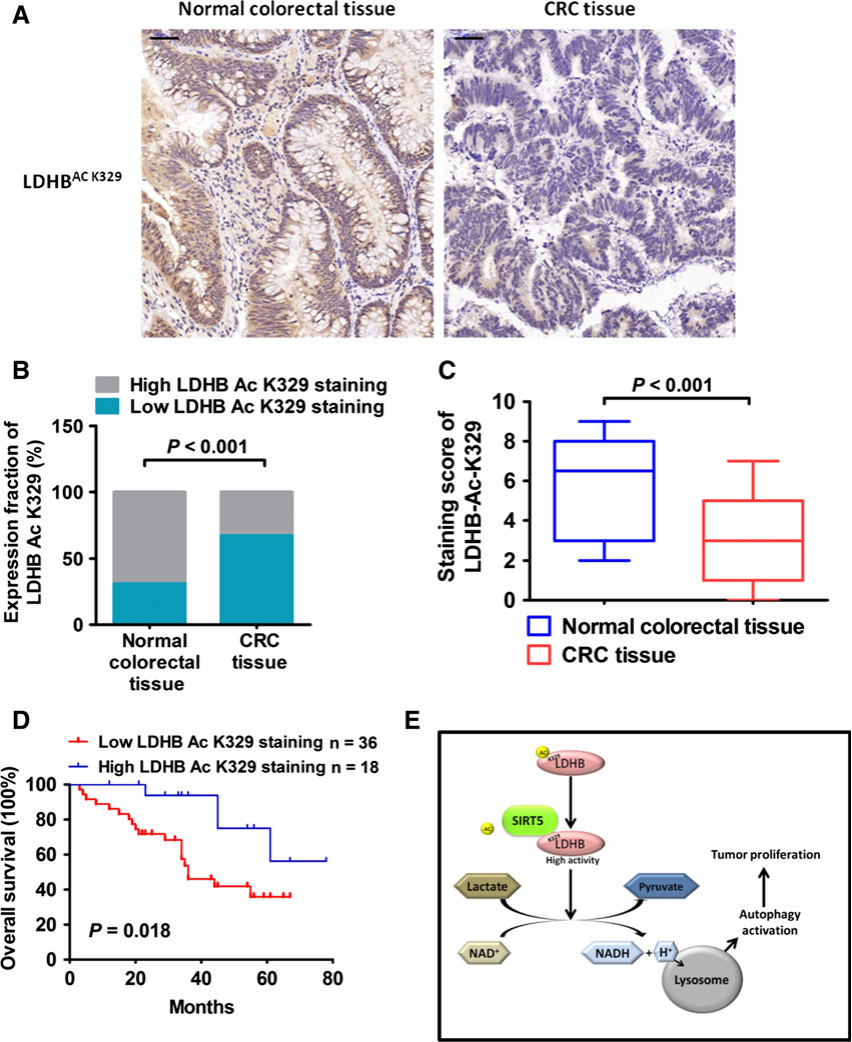
Decreased LDHB K329 acetylation in human CRC samples correlates with poor prognosis. (A) Representative bright‐field images showing LDHB‐Ac‐K329 in human colorectal cancer sections and adjacent normal tissue. Scale bar, 50 μm. (B) Statistical analysis was conducted with the chi‐square test based on the amount of high or low LDHB‐Ac‐K329 staining in the peritumour/tumour cases. (C) The mean LDHB‐Ac‐K329 staining scores in CRC tissues and normal peritumoral tissues. (D) The Kaplan–Meier analysis of overall survival depending on the LDHB‐Ac‐K329 level in CRC tumour tissues. (E) Working model of the role of SIRT5 in regulating LDHB‐mediated autophagy and colorectal cancer proliferation.

The finding that the deacetylation of LDHB by SIRT5 promotes autophagy and cancer cell proliferation led us to examine K329 acetylation status in human CRC tissues. A total of 54 pairs of CRC samples with adjacent normal colorectal tissues were examined by immunostaining with antibody against LDHB acetylated at K329 (LDHB‐Ac‐K329; Fig. 6A). High K329 acetylation was detected in 69% (37 of 54) of normal tissues, and only 33% (18 of 54) of tumour tissues exhibited high K329 acetylation status (*P* < 0.001; Fig. 6B). The mean LDHB‐Ac‐K329 staining score in CRC tissues was lower than that in corresponding normal tissues (*P* < 0.001; Fig. 6C). Univariate statistical analysis found that LDHB‐Ac‐K329 levels were negatively associated with tumour size (*P* = 0.004) and histological grade (*P* = 0.031; Table 1). Finally, we found that low LDHB‐Ac‐K329 staining predicted poor overall patient survival (Fig. 6D). These results suggest that LDHB K329 acetylation status might be used to predict tumour progression in human CRC.

**Table 1:** Relationship between LDHB‐Ac‐K329 level and clinicopathological parameters in patients with colorectal cancer

Characteristics	LDHB‐Ac‐K329 level			*P*‐value
	All cases	Low	High	
Participants	54	36	18	
Age				
<60 years	23	17	6	0.331
≥60 years	31	19	12	
Gender				
Male	32	22	10	0.695
Female	22	14	8	
Tumour stage				
0–II	33	21	12	0.554
III–IV	21	15	6	
Tumour size (cm)				
<4.0	24	11	13	0.004
≥4.0	30	25	5	
Histological grade				
Moderately and highly differentiated	22	11	11	0.031
Poorly differentiated	32	25	7	
Lymph node metastasis				
Negative	33	21	12	0.554
Positive	21	15	6	

## 4 Discussion

Lactate dehydrogenase B, catalysing the conversion of lactate and NAD^+^ to pyruvate, NADH and H^+^, is a key contributor to lysosomal acidification and autophagy in cancer (Brisson *et al*., 2016). LDHB is highly expressed in multiple types of cancer, including medulloblastoma, cholangiocarcinoma, oesophageal squamous cell carcinoma and breast cancer (de Haas *et al*., 2008; Isozaki *et al*., 2012; McCleland *et al*., 2012; Yoo *et al*., 2009). Furthermore, elevated LDHB expression had a significant impact on cancer progression and metastasis (Koshiyama *et al*., 2013; Li *et al*., 2016; McCleland *et al*., 2012). Therefore, LDHB has an important role in cancer progression. PTM regulates the function of multiple metabolic enzymes and the consequent cellular biochemical metabolism and biological status. Interestingly, our data proved that LDHB is an acetylated protein and verified that K329 of LDHB is the major deacetylation residue modified by SIRT5. Although functional LDH is a homo‐ or heterotetramer composed of LDHA and LDHB subunits, LDHA does not harbour a motif comparable to K329 in LDHB and our research proved that SIRT5 only regulated LDHB activity but not LDHA. Thus, in this study, we uncovered a mechanism of LDHB regulation that contributes to its increased activity by SIRT5 on R329 in CRC cells.

SIRT5 is a member of the sirtuin family, which can sense nutrient requirements and optimize cellular pathways to maintain metabolic homoeostasis and cellular survival under limited nutritional conditions (Guedouari *et al*., 2017). Recent studies have revealed that SIRT5 is involved in tumorigenesis via its PTM activities (Wang *et al*., 2018; Xiangyun *et al*., 2017). In our study, when we used Flag‐SIRT5 as the bait to find its possible partners, LDHB was identified from the SIRT5 interaction network. We further found that SIRT5 regulates autophagy, apoptosis and proliferation of CRC cells via its association with LDHB. The deacetylation of LDHB by SIRT5 powerfully enhanced LDHB enzymatic activity. However, SIRT5 has also been reported as a suppressor of autophagy in the human breast cancer cell line MDA‐MB‐231 (Polletta *et al*., 2015), indicating that SIRT5 might act as both autophagy promoter and suppressor in a context‐dependent manner. Notably, MCT1, which facilitates lactate uptake, is not expressed in MDA‐MB‐231 (Hussien and Brooks, 2011). Lactate is the metabolic substrate of LDHB. In addition, MDA‐MB‐231 cells expressed mainly LDHA, not LDHB (Hussien and Brooks, 2011). Therefore, the function of SIRT5 in autophagy is context‐dependent.

Lactate dehydrogenase B interacts with V‐ATPase at the lysosomal surface; as a result, the protons (H^+^) generated by LDHB promote lysosomal acidification. Lysosomal acidification is important for the following vesicle maturation and protease activation during autophagy. In our study, LDHB acetylation or SIRT5 KO prevented the lysosomal acidification and decreased the abundance of mature autolysosomes. LDHB acetylation or SIRT5 KO also induced leupeptin‐sensitive LC3‐II protein accumulation, which represented a potent inhibition of the autophagic flux (Rubinsztein *et al*., 2009) and prevented degradation of autophagic substrate optineurin. Thus, it was reasonable to believe that SIRT5‐deacetylated LDHB could promote the lysosomal acidification and, subsequently, autophagy, which are essential for cancer development (Fig. 6E).

It has been shown that inhibiting autophagy with agents, such as chloroquine, may lead to cell death, which implies a promising therapeutic approach against cancer (Maclean *et al*., 2008; Morgan *et al*., 2014). The modulation of the key elements of the autophagy pathway is also being exploited as a novel therapeutic option for CRC treatment (Mokarram *et al*., 2017). The deletion of the autophagy‐related gene may prevent the development and progression of CRC in genetically predisposed patients (Levy *et al*., 2015). In another study on human CRC cells, inhibiting autophagy increases anticancer drug‐induced apoptotic cell death (Gurkan *et al*., 2018). Thus, the promotion of autophagy by SIRT5 via LDHB suggested that they might affect cancer growth. Besides being an important regulator in autophagy, LDHB oxidates lactate to pyruvate, which fuels the TCA cycle and benefits for oxidative cancer cells (Sonveaux *et al*., 2008). Our results showed that LDHB K329 deacetylation significantly increased cell respiration and ATP generation in oxidative HCT116 cells. These findings imply that cell respiration mediated by LDHB deacetylation might also contribute to cancer cell growth. In this study, our data show that SIRT5 KO or LDHB knockdown inhibited proliferation of CRC cells. Moreover, SIRT5‐mediated regulation of cell proliferation was dependent on the deacetylation of LDHB at the K329. Furthermore, our in vivo growth assays showed that cancer cells overexpressing LDHB^WT^ grew quicker than cells overexpressing LDHB^K329Q^, whereas the growth advantage induced by LDHB^WT^ was blocked by the presence of SIRT5 inhibitor GW5074. Although GW5074 could impair clonogenic and tumorigenic capacity of CRC cells by its inhibitory effect on c‐Raf kinase activity (Borovski *et al*., 2017), our studies demonstrated it could significantly reduce the expression level of endogenous SIRT5 and blocked autophagic flux and tumour cell proliferation via SIRT5/LDHB axis. Considering the side effects of lysosomotropic agent chloroquine (Maycotte *et al*., 2012) and a decrease in chloroquine activity by tumour acidity (Pellegrini *et al*., 2014), SIRT5‐mediated LDHB deacetylation pathway might be a new therapeutic target for treating CRC. In addition, we found that LDHB K329 acetylation was lower in CRC tissues than in normal colorectal tissues. Significantly, low levels of LDHB‐Ac‐K329 in tumours may predict short survival time in patients with CRC. Hence, the LDHB‐Ac‐K329 status is a potential prognostic indicator for patients with CRC and might be useful for identifying the patients with CRC who are suitable for anti‐autophagy treatment.

## 5 Conclusions

Overall, these results suggested a model where SIRT5 directly interacts with LDHB and yields strong deacetylation of LDHB at the K329 site. This effect increases LDHB activity and favours lysosomal acidification and autolysosomal maturation. Enhanced autophagy along with reduced apoptotic cell death contributes to colorectal tumour progression. This confirms that targeting SIRT5/LDHB pathway might be a feasible and effective strategy in the treatment of CRC.

## Conflict of interest

The authors declare no conflict of interest.

## Author contributions

JL, GH, LS and HY designed the project; LS, HY, LZ, SA, MS, RZ and WJ performed the experiments; LS and HY analysed and interpreted the data; HY and LZ contributed materials; JL, GH, LS and HY prepared the manuscript; and all authors read and approved the final manuscript.

## Supporting information


**Fig. S1.** SIRT5 does not affect the succinylation, malonylation or glutarylation of LDHB.
**Fig. S2.** SIRT5 increases LDHB activity.
**Fig. S3.** SIRT5 promotes autophagy via LDHB deacetylation.
**Fig. S4.** LDHB deacetylation promotes cell respiration.
**Fig. S5.** LDHB deacetylation promotes tumour growth in vivo.
