# Supplementary material for: SIRT5‐mediated deacetylation of LDHB promotes autophagy and tumorigenesis in colorectal cancer
Source: Mol Oncol. 2018 Dec 3;13(2):358–75. doi: 10.1002/1878-0261.12408 (PMC6360364; doi:10.1002/1878-0261.12408)
Supplement: Supplementary file 1 — Fig. S1. SIRT5 does not affect the succinylation, malonylation or glutarylation of LDHB. Fig. S2. SIRT5 increases LDHB activity. Fig. S3. SIRT5 promotes autophagy via LDHB deacetylation. Fig. S4. LDHB deacetylation promotes cell respiration. Fig. S5. LDHB deacetylation promotes tumour growth in vivo. [file MOL2-13-358-s001.pdf]

Supplementary figures and legends:

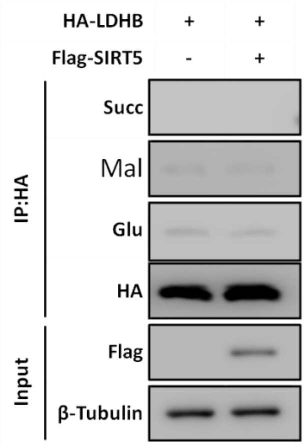

Fig. S1. SIRT5 does not affect the succinylation, malonylation or glutarylation of LDHB. HCT116 cells were transfected with HA-LDHB and Flag-SIRT5 (or empty vector as a control) for 48 h. Cell lysates were immunoprecipitated with HA-LDHB affinity gel. LDHB succinylation (Succ), malonylation (Mal), glutarylation (Glu) and protein levels were analysed by immunoblotting using the indicated antibodies. Relative ratios of acetylation were calculated from normalizing against HA-LDHB.

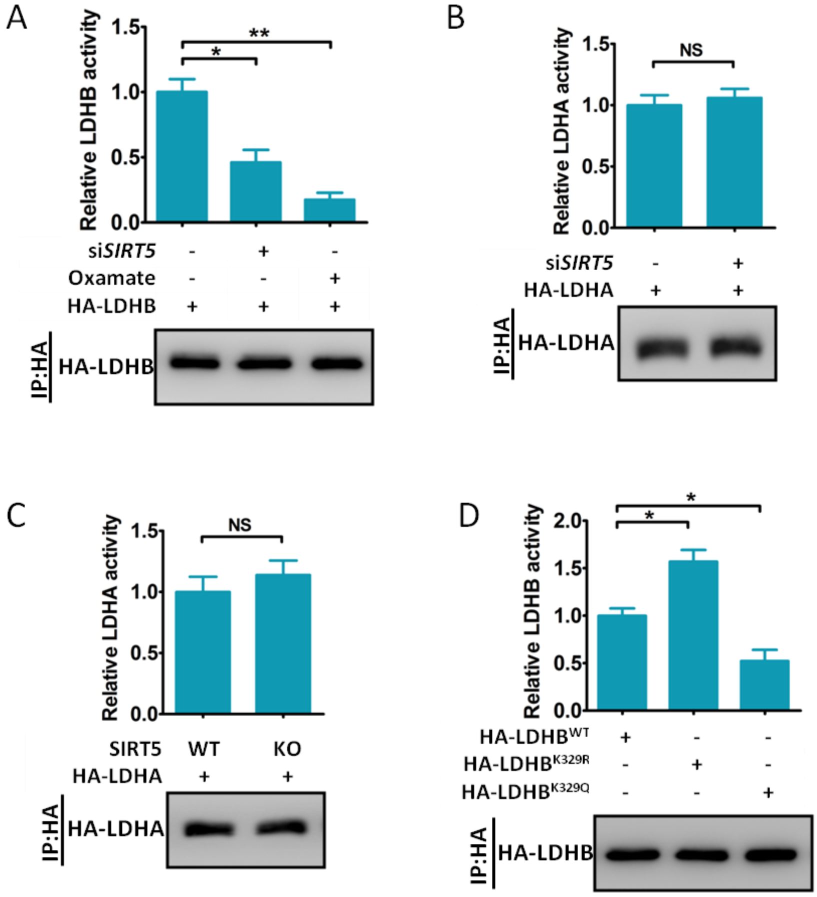

Fig. S2. SIRT5 increases LDHB activity. (A) DLD1 cells were transfected with HA-LDHB and siRNA against SIRT5 or negative control siRNA for 48 h. In addition, control cells were treated with the LDHB inhibitor oxamate (10 mM) for the last 24 h. HA-LDHB was immunoprecipitated, and LDHB activity was assayed. LDHB activity was normalised against protein levels. Error bars represent means ± SEM (n=3). (B) HCT116 cells were transfected with HA-LDHA and siRNA against SIRT5 or negative control siRNA for 48 h. HA-LDHA was immunoprecipitated, and LDHA activity was assayed. LDHA activity was normalised against protein levels. Error bars represent means ± SEM (n=3). (C) SIRT5 WT/KO HCT116 cells were transfected with HA-LDHA for 48 h. LDHA was immunoprecipitated, and LDHA activity was assayed. LDHA activity was normalized against protein levels. Error bars represent means ± SEM (n=3). (D) HA-tagged wild-type and mutant LDHB proteins were expressed in DLD1 cells and purified by immunoprecipitation. The enzyme activity was measured and normalised against the protein level. Relative enzyme activities of triplicate experiments with ± SEM are presented (n=3). **P* < 0.05, ***P* < 0.01. *P* values were based on Student's t test.

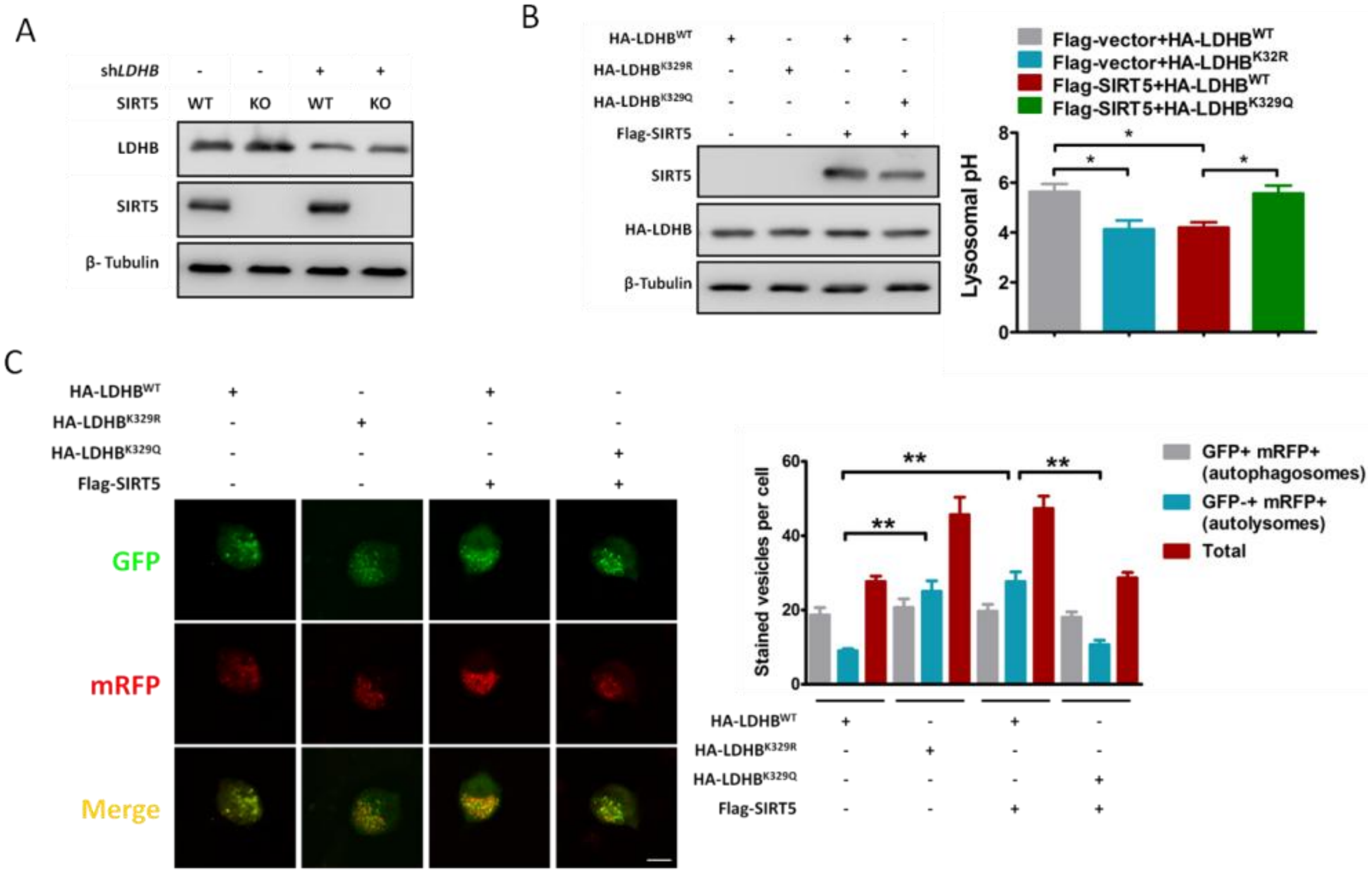

Fig. S3. SIRT5 promotes autophagy via LDHB deacetylation. (A) LDHB was stably knocked down in DLD1 SIRT5 KO cells. LDHB, SIRT5 and β-Tubulin levels were determined by immunoblotting. (B-C) SIRT5 KO and sh*LDHB* DLD1 cells were transfected with HA-LDHB^{WT}, HA-LDHB^{K329R}, HA-LDHB^{WT}+Flag-SIRT5, or HA-LDHB^{K329Q}+Flag-SIRT5. (B) Lysosomal pH was measured with FITC-dextran. Error bars represent means ± SEM (n=3). (C) Abundance of autophagosomes and of autolysosomes. Scale bar, 10 μm. Error bars represent means ± SEM (n=3). *$P < 0.05$, **$P < 0.01$. *P* values were based on Student's t test.

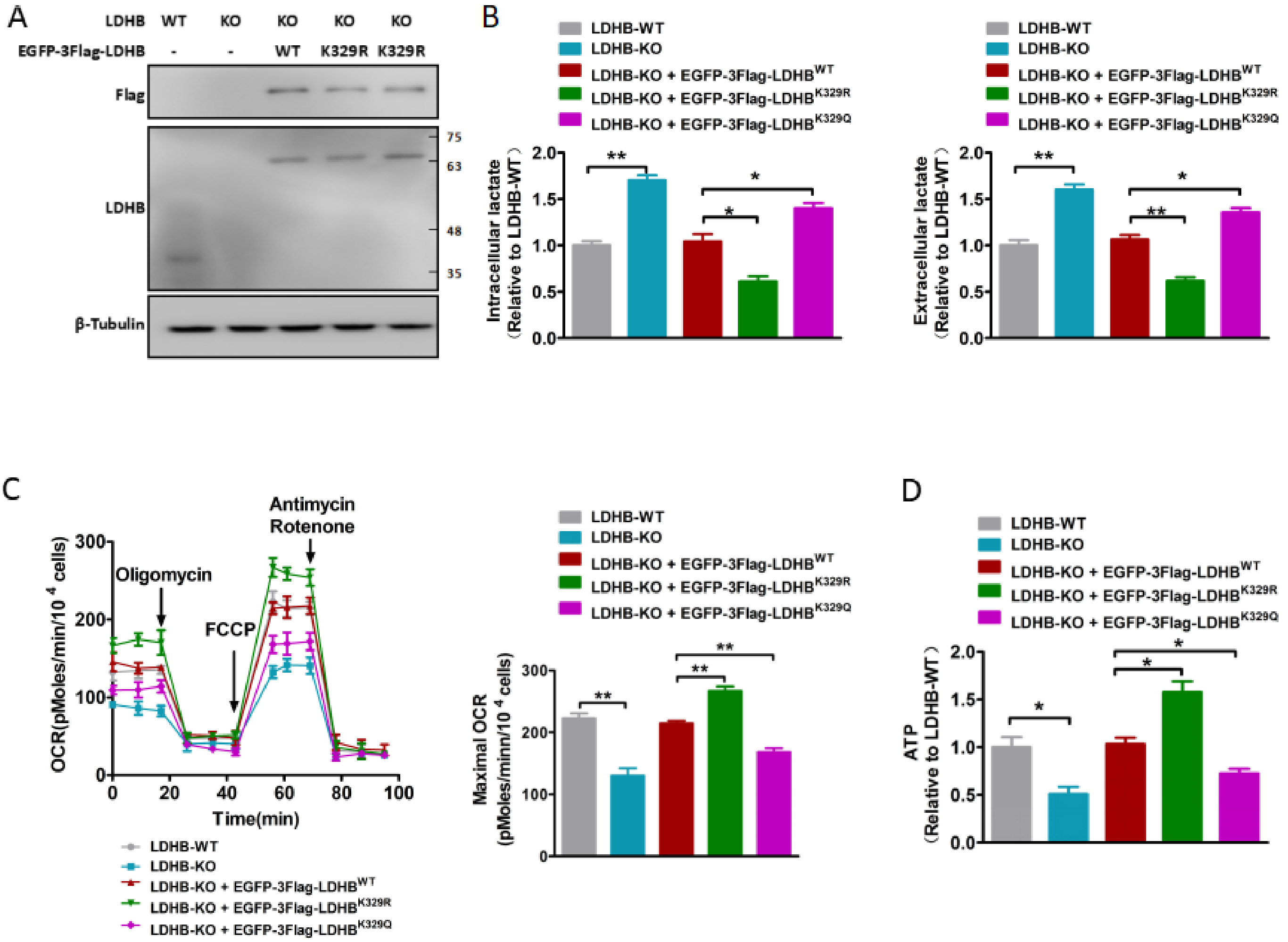

Fig. S4. LDHB deacetylation promotes cell respiration. (A-D) LDHB KO HCT116 cells were infected with lentivirus suspension containing EGFP-3Flag-LDHB^{WT}/EGFP-3Flag-LDHB^{K329R}/EGFP-3Flag-LDHB^{K329Q}. LDHB re-expression was determined by western blotting (A). Lactate levels in medium and in cells were examined and normalized by cell numbers. Error bars represent means ± SEM (n=3) (B). 1×10^4 cells were plated into appropriate plates for analysis of basal oxygen consumption rate (OCR) by a Seahorse XF24 extracellular flux analyzer. Error bars represent means ± SEM (n=3) (C). Cellular ATP levels were quantitated by the colorimetric method and normalized by cell numbers. Error bars represent means ± SEM (n=3) (D). **P* < 0.05, ***P* < 0.01. *P* values were based on Student's t test.

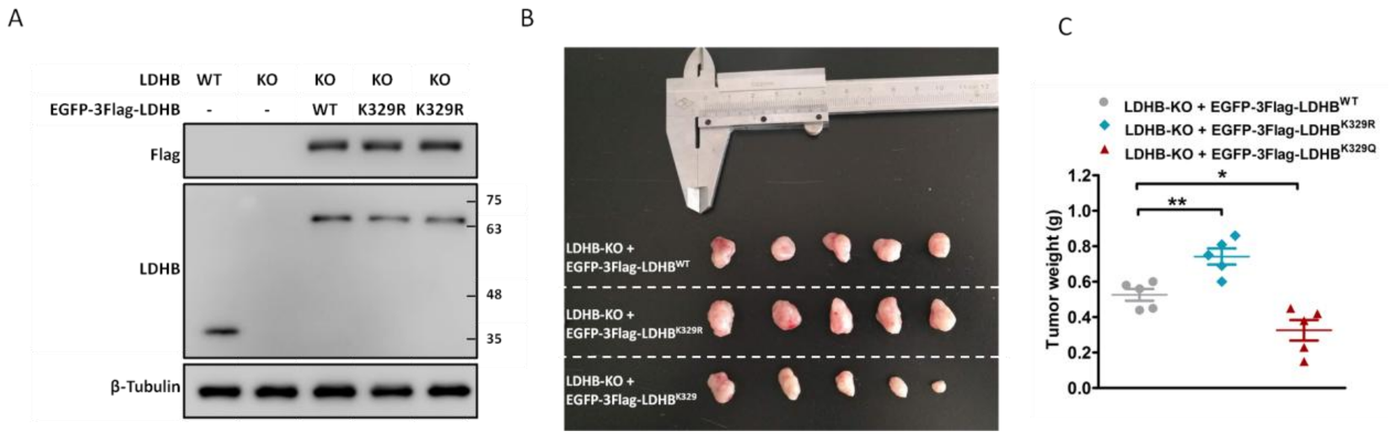

Fig. S5. LDHB deacetylation promotes tumour growth in vivo. (A) LDHB KO DLD1 cells were infected with lentivirus suspension containing EGFP-3Flag-LDHB^{WT}/EGFP-3Flag-LDHB^{K329R}/EGFP-3Flag-LDHB^{K329Q}. LDHB re-expression was determined by western blotting. (B) A total of 5 × 10^6 LDHB KO DLD1 cells stably expressing EGFP-3Flag-LDHB$^{WT/K329R/K329Q}$ were injected subcutaneously into the right dorsal flank of nude mice. Image of tumours isolated from nude mice. (n = 5 for each group) (C) The weight of tumours when mice were sacrificed. Error bars represent means ± SEM (n=5). *P < 0.05, **P < 0.01. P values were based on Student's t test.
